# Brain Atrophy and White Matter Damage Linked to Peripheral Bioenergetic Deficits in the Neurodegenerative Disease FXTAS

**DOI:** 10.3390/ijms22179171

**Published:** 2021-08-25

**Authors:** Junyi Wang, Eleonora Napoli, Kyoungmi Kim, Yingratana A. McLennan, Randi J. Hagerman, Cecilia Giulivi

**Affiliations:** 1 Center for Mind and Brain, University of California Davis, Davis, CA 95618, USA; jyiwang@ucdavis.edu; 2 Department of Molecular Biosciences, School of Veterinary Medicine, University of California Davis, Davis, CA 95616, USA; enapoli@ucdavis.edu; 3 The MIND Institute, University of California Davis Medical Center, Sacramento, CA 95817, USA; kmkim@ucdavis.edu (K.K.); yamclennan@ucdavis.edu (Y.A.M.); 4 Department of Public Health Sciences, School of Medicine, University of California Davis, Sacramento, CA 95817, USA; 5 Department of Pediatrics, University of California Davis Medical Center, Sacramento, CA 95817, USA

**Keywords:** aging, cognition, brain, MRI, volume, white matter hyperintensities, mitochondria, bioenergetics, peripheral blood monocytic cells, *FMR1*

## Abstract

Fragile X-associated tremor/ataxia syndrome (FXTAS) is a neurodegenerative disorder affecting subjects (premutation carriers) with a 55-200 CGG-trinucleotide expansion in the 5′UTR of the fragile X mental retardation 1 gene (*FMR1*) typically after age 50. As both the presence of white matter hyperintensities (WMHs) and atrophied gray matter on magnetic resonance imaging (MRI) are linked to age-dependent decline in cognition, here we tested whether MRI outcomes (WMH volume (WMHV) and brain volume) were correlated with mitochondrial bioenergetics from peripheral blood monocytic cells in 87 carriers with and without FXTAS. As a parameter assessing cumulative damage, WMHV was correlated to both FXTAS stages and age, and brain volume discriminated between carriers and non-carriers. Similarly, mitochondrial mass and ATP production showed an age-dependent decline across all participants, but in contrast to WMHV, only FADH_2_-linked ATP production was significantly reduced in carriers vs. non-carriers. In carriers, WMHV negatively correlated with ATP production sustained by glucose-glutamine and FADH_2_-linked substrates, whereas brain volume was positively associated with the latter and mitochondrial mass. The observed correlations between peripheral mitochondrial bioenergetics and MRI findings—and the lack of correlations with FXTAS diagnosis/stages—may stem from early brain bioenergetic deficits even before overt FXTAS symptoms and/or imaging findings.

## 1. Introduction

The onset of neurodegenerative diseases such as Alzheimer’s disease (AD), Parkinson’s disease (PD), Huntington’s disease (HD), fragile X-associated tremor/ataxia syndrome (FXTAS), among others, is believed to be multifactorial; however, older age is the greatest risk factor [1,2,3]. Thus, it is likely that cellular and molecular changes associated with aging and/or premature senescence would promote neuronal abnormalities and degeneration. Among them, perturbation of cellular energy metabolism and mitochondrial biogenesis are commonly associated with aging. These changes in energy metabolism have been partly explained by decreased sensitivity to glucose signaling, uptake, and utilization [4] resulting in major metabolic disorders, which are also well-known contributors to neurodegeneration.

Given that the brain is ~2% of the body weight but accounts for 20% of the energy consumption in humans [5,6], it is not surprising that a decline in mitochondrial bioenergetics often results in neuropsychiatric deficits [7]. The brain’s high energy demand is met mainly by mitochondria-dependent glucose metabolism [8]. As such, neurons are the cells most vulnerable to reduced glucose supply due to their high energy demand. Energy failure is followed by an imbalance in the redox status and hyperexcitability, neuronal necrosis or apoptosis with dire consequences to brain trauma and neurodegeneration [9]. The mechanisms of age- and disease-dependent impaired energy production in the brain that significantly lower cognitive function and increase the risk for developing neurodegeneration are yet to be identified [10]. For instance, a significant reduction of glucose uptake in the brain is detected in prodromal [11,12,13,14] as well as symptomatic [15,16,17] stages of familial AD, suggesting that abnormalities in neuronal energy metabolism and subsequent energy depletion are the earliest pathophysiological conditions prior to the onset of overt clinical manifestations of AD. The same phenomenon has also been observed in other neurodegenerative diseases such as HD and FXTAS [18,19,20,21,22,23,24]. Consistent with this premise, mitochondrial dysfunction, impairment of oxidative phosphorylation, and reduced glucose metabolism have been shown to be early pathological alterations in AD [25,26]. Concomitantly to energy depletion, both reduced glucose uptake and mitochondrial dysfunction may cause a robust generation of reactive oxygen species (ROS), which would be an additional insult leading to accelerated damage in neurons [27,28,29], as oxidative damage is a known contributor to neurodegenerative processes [30,31,32,33].

Indeed, several dietary and pharmacological interventions have been designed to modulate cellular metabolism with anti-aging purposes as well as for slowing the progression of neurodegeneration. Among these, caloric restriction [34,35,36,37], administration of resveratrol [38] and metformin [39], and electroacupuncture [40] have recently emerged as promising therapeutic strategies with a common underlying mechanism, specifically the activation of SIRT1 and/or AMPK pathways and inhibition of mTOR signaling, leading to a slowing of the aging process and a decrease of the incidence of age-related neurodegeneration [41,42,43].

Our team was the first to report mitochondrial dysfunction as a common feature in biological samples including primary skin fibroblasts, peripheral blood monocytic cells (PBMCs), serum/plasma and postmortem brain tissues from carriers of the *FMR1* premutation (defined as a moderate (55–200) CGG repeat expansion in the *FMR1* gene) with and without FXTAS [23,24,44,45,46] as well as in murine models of the premutation [47]. However, to our knowledge, no study to date has characterized the putative correlation between biomarkers of neuronal or brain aging, associated with cognitive decline, and peripheral mitochondrial bioenergetic status. Only one other study reported a positive correlation between brain white matter hyperintensities (WMHs) scored from MRI and improved mitochondrial outcomes in Epstein-Barr virus (EBV)-transformed lymphoblasts in carriers of the premutation with and without FXTAS [48]. Such correlation may have resulted from the use of EBV-transformed lymphoblastoid cell lines (lymphoblast cell line or LCL). Despite the fact that cultured fibroblasts and LCL have been found to be extremely useful in the diagnosis of mitochondrial disorders [49,50], both systems have the disadvantage that the mitochondrial defect may or may not be expressed to the level that matches those of the primarily affected organ or tissue. Dissimilarities between these cell types rely on the fact that values for respiratory chain-deficient LCL are not nearly as elevated as they are for cultured skin fibroblasts. Because LCL are a transformed cell line, they do not go into the semiquiescent state of confluence, and have a constantly high ATP demand that tends to keep the redox states in a rather oxidized condition, confounding the detection of high lactate-to-pyruvate ratios for respiratory chain defects [49]. In addition, upon EBV transformation, LCL have been found to have higher mitochondrial biogenesis than lymphocytes [51], loss of methylation near the trinucleotide expansion of the myotonic dystrophy protein kinase gene [52], and higher formation of complex mtDNA arrangements [53], among other issues [54,55,56,57,58,59]. 

The presence and early detection of mitochondrial dysfunction, before the onset of overt clinical symptoms, would allow physicians to detect early signs of aging or premature aging in individuals at risk of developing neurodegeneration. This concept is based on the difference between the chronological age (based on the date of birth) and the biological one (internal clock). To address this gap in knowledge in the context of the neurodegenerative disease FXTAS, we sought to correlate brain volume (BV) and white matter lesions quantified using the WMH volumes (WMHV)—which were already reported as affected in carriers of the premutation [60]—with mitochondrial outcomes from terminally differentiated, non-proliferating peripheral blood mononuclear cells (PBMCs).

Our study indicates the feasibility of correlating MRI imaging with some peripheral bioenergetic markers, thereby providing the potential to identify those cases with early signs of premature aging and a potentially worse prognosis.

## 2. Results

### 2.1. Demographic Characteristics of Participants Included in This Study

This study enrolled 103 individuals (16 non-carriers, mean age (SD) 50 (18) years, range 24–73 years; 87 premutation carriers, mean age (SD) 63 (12) years, range 24–85 years) with available MRI studies and bioenergetic analyses of PBMCs except for 7 non-carriers who had only mitochondrial function assessment. About half of the participants were female (non-carriers: 4/16 (25%), premutation carriers: 42/87 (48%)). Premutation carriers were grouped by FXTAS stage, which was scored by a trained physician (RJH) based on the severity of movement and gait impairments (stage 1: subtle or questionable signs, stages 2–6: clear tremor/balance problems with minor to severe interference of daily living; [61]). Premutation carriers at FXTAS stage ≤ 1 were included under the PFX-group (N = 24, 6 males and 18 females, mean age (SD) 49 (13) years, range 24–71 years) whereas those at the FXTAS stages ≥ 2 were grouped into PFX+ (N = 63, 39 males and 24 females, mean age (SD) 69 (8) years, range 53–85 years).

### 2.2. Smaller Brain Volume in Premutation Carriers Compared to Non-Carriers, and Higher WMHV in Carriers with FXTAS Stages ≥ 2

**Figure 1:**
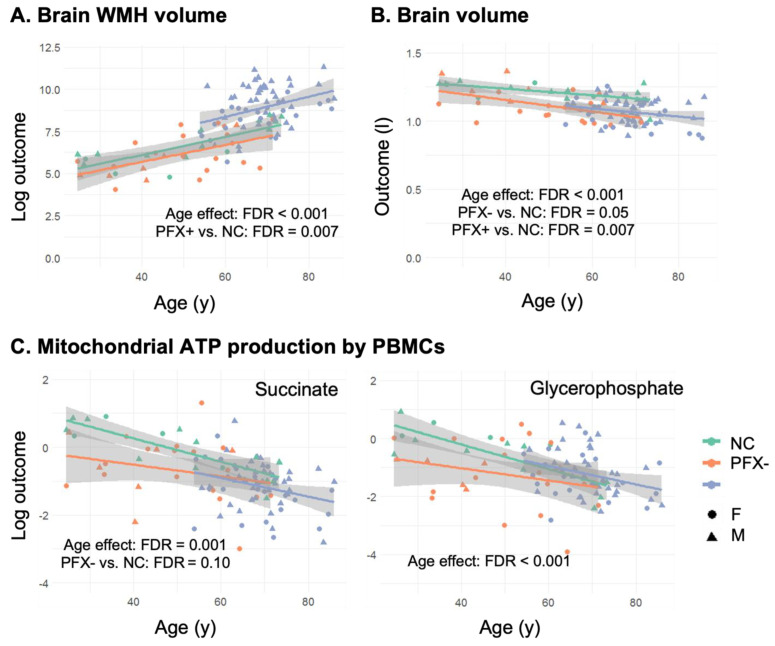
Group differences and correlations between age, brain volumes and mitochondrial outcomes in premutation carriers at FXTAS stages 0 and 1 (PFX−), FXTAS stages 2–5 (PFX+), and non-carriers (NC) (**A**) White matter hyperintensity (WMH) volume. (**B**) Whole brain volume. (**C**) Peripheral mitochondrial ATP production by PBMCs sustained by succinate (**left** panel) and glycerophosphate (**right** panel).

Multiple linear regression analysis was conducted to examine the effects of age, group (i.e., FXTAS status), sex, and age-by-group interaction on individual MRI outcomes. Brain scaling factor was included as a covariate for MRI outcomes to control individual differences in cranial size. The regression analyses revealed significant correlations between age and WMHV (directly) (β = 0.05 ± 0.011 log(mL), false discovery rate (FDR) < 0.001) and between age and BV (inversely) (β = −3.18 ± 0.62 mL, FDR < 0.001), while controlling for sex and group. There was also a sex difference in WMHV indicating that females had smaller WMHV than males (females = 3.32 ± 3.83 mL, males = 13.3 ± 16.9 mL, FDR = 0.01). However, in a post-hoc analysis including premutation carriers only in the regression analysis, the sex-differences in WMHV were not significant after adding FXTAS stage as a covariate (*p* = 0.06). Compared with non-carriers, PFX− exhibited smaller BV (β = −52.2 ± 24.4 mL, FDR = 0.05) with no difference in WMHV (β = 0.005 ± 0.45 log(mL), FDR = 0.99) after adjusting for age and sex. In contrast, PFX+ exhibited significantly higher WMHV and smaller BV compared with both non-carriers (β_WMHV_/β_BV_ = 1.54 ± 0.52 log(mL)/−86.8 ± 28.6 mL, both FDRs = 0.007) and PFX− (β_WMHV_/β_BV_ = 1.53 ± 0.36 log(mL)/−34.7 ± 19.6 mL, FDR < 0.001/0.09; Table 1, Figure 1A,B).

### 2.3. Lower Overall Oxygen-Linked ATP Production Fueled by Succinate in Premutation PBMCs but Higher with Glycerophosphate as Substrate in Carriers with FXTAS Stages ≥ 2

Multiple linear regression analysis was conducted to examine the effects of age, group, sex, and age-by-group interaction on individual mitochondrial outcomes. Of the 11 mitochondrial outcomes tested in PBMCs, 6 (all log transformed) revealed significant age-dependent decreases across the 3 diagnostic groups (β = −0.035 to −0.021, FDR < 0.001–0.008). Among these were two markers of mitochondrial mass (citrate synthase, and cytochrome *c* oxidase or Complex IV activities), oxygen-linked ATP production by various segments of the electron transport chain sustained by NADH-, FADH_2_-, and alpha-glycerophosphate-linked substrates; and oxygen-linked ATP production fueled by glucose and glutamine (Table 1).

Oxygen-linked ATP production sustained by succinate (FADH_2_-linked respiration), was lower in carriers, regardless of FXTAS diagnosis, compared to non-carriers after age-adjustment (PFX− vs. non-carriers/PFX+ vs. non-carriers: β = −0.57/−0.55, FDR = 0.10) (Figure 1C, left panel). ATP production sustained by alpha-glycerophosphate was higher in PFX+ as a group compared to PFX− after age-adjustment (β = 0.64, FDR = 0.10; Figure 1C, right panel) suggesting either a decreased activity of the malate-aspartate shuttle or increased fatty acid oxidation to sustain the ATP demand. No sex differences were observed for peripheral mitochondrial outcomes in non-carriers. Unexpectedly, due to the X-linked nature of the disorder, no significant sex differences were observed for the carriers either.

### 2.4. Age-Dependent Negative Correlation between WMHV and Oxygen-Linked ATP Production, and Positive Correlation between Brain Volume and ATP Production and Mitochondrial Mass in the Premutation Carriers

**Figure 2:**
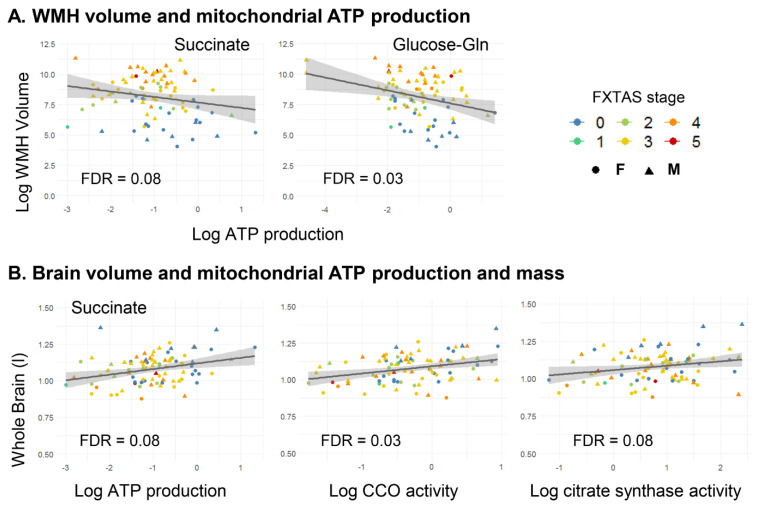
Correlation between peripheral mitochondrial outcomes and volumes of WMHs and whole brain in premutation carriers. (**A**) Correlations between WMHV and mitochondrial ATP production sustained by succinate (**left** panel) and glucose-Gln (**right** panel). (**B**) Correlations between whole brain volume and mitochondrial ATP production sustained by succinate and mitochondrial mass (CCO and citrate synthase activities). Two outliers for glucose-Gln sustained ATP production and one outlier for CCO activity (with very small values) were removed from the analyses. CCO, cytochrome *c* oxidase.

Semi-partial correlations between mitochondrial outcomes and MRI data (while controlling for cranial size) were tested in the cohort constituted by the 87 premutation carriers. Among the 11 mitochondrial outcomes, ATP production fueled by succinate and glucose-glutamine showed significant negative correlations with brain WMHV (FADH_2_-dependent oxygen consumption/basal r = −0.26/−0.33, FDR = 0.08/0.03). Both FADH_2_-dependent ATP production and markers of mitochondrial mass, namely citrate synthase and Complex IV activities, were correlated positively with BV (r = 0.25–0.32, FDR = 0.03–0.08; Table 2, Figure 2). More importantly, no differences with sex were observed. When age was included as a covariate, all correlations between mitochondrial outcomes and MRI data were no longer significant, indicating age as the main driver of the correlations between MRI findings and peripheral mitochondrial outcomes.

**Figure 3:**
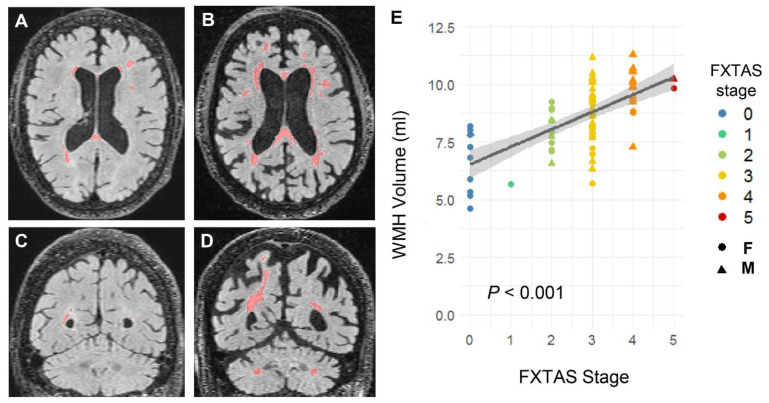
WMH quantifications and correlation with FXTAS stage. (**A**) Representative axial view of the FLAIR image showing WMHs in the periventricular regions and deep white matter for a 68-y-old female carrier at FXTAS stage 3. (**B**) Representative axial view of the FLAIR image showing more extensive WMHs in the periventricular regions and deep white matter for a 69-y-old male carrier at FXTAS stage 3 relative to the female carrier shown in panel (**A**). (**C**) A coronal view of the FLAIR image showing periventricular WMHs at the posterior horn of the lateral ventricles for the same female carrier as it is in (**A**). (**D**) A coronal view of the FLAIR image showing periventricular WMHs extending to the white matter in the right parietal lobe as well as bilateral WMHs in the middle cerebellar peduncle in the same male carrier as in (**B**). (**E**) Correlation between WMHV and FXTAS stage in male and female carriers aged 50 years and older.

Consistent with the above results, and with the premise that age is considered the greatest risk factor in neurodegenerative disorders [1,2,3] and FXTAS mainly affects premutation carriers older than 50 years, the correlations between FXTAS stage (as proxy for the impact of tremor and ataxia has on daily activities [61]) and MRI/mitochondrial measurements were analyzed with only those carriers aged 50 y and above (N = 74, 40 males, 34 females). In agreement with the concept that increases in WMHV are core features of FXTAS pointing to cumulative brain damage, only WMHV, adjusted for age, sex, and cranial size, showed a significant correlation with FXTAS stage (β = 0.42 ± 0.097, *p* < 0.001; Figure 3). None of the mitochondrial outcomes or BV showed significant correlations with FXTAS stage (*p* > 0.15; Supplementary Table S1).

## 3. Discussion

WMHs in the middle cerebellar peduncle and cerebral white matter, along with generalized brain atrophy, are core radiological features of FXTAS and, as such, used as criteria for FXTAS diagnosis [62,63]. Mitochondrial dysfunction, demonstrated in several biological samples of premutation carriers with and without FXTAS, has been recognized as an early marker of *FMR1* premutation even without overt signs of clinical symptoms [18,19,21,22,64]. Here, for the first time, we examined the putative correlations of WMHV and BV with several outcomes of peripheral mitochondrial bioenergetics between *FMR1* premutation carriers and non-carriers in a relatively large cohort (87 carriers vs. 16 non-carriers).

In the context of MRI findings, both WMHV and BV correlated with FXTAS morbidity as WMHV was significantly higher and whole BV was lower in PFX+ (at FXTAS stages ≥ 2) compared with both PFX− (at FXTAS stages ≤ 1) and non-carriers (after adjusting for age, sex, and cranial size; FDR < 0.001–0.09). Only BV was smaller in PFX− (FDR = 0.05) compared with non-carriers (Table 2, Figure 1B). The smaller BV in PFX− relative to non-carriers is consistent with our previous cross-sectional study using a larger dataset of males (142 non-carriers and 109 PFX, aged 8–81 years), which reported accelerated BV decrease in PFX− compared with non-carriers [65]. We also showed significant changes in WMHV and BV with age across all participants (FDR < 0.001) in agreement with other studies [66,67,68,69,70].

WMHV was also lower in females than males (FDR = 0.01) regardless of the premutation status. However, in a post-hoc analysis including only premutation carriers, this significance was no longer observed after adding FXTAS stage as a covariate, suggesting that WMHV is mainly impacted by FXTAS progression. These findings are consistent with an early study that reported increased WMHV in PFX+ compared with both PFX− and non-carriers of the same sex but, contrary to our findings, no differences in WMHV were identified between sexes [71]. This discrepancy may originate from the uneven sex distribution of carriers across FXTAS stages, females being more numerous than males at lower FXTAS stages (75% females vs. 25% males at stages 0 and 1) and males being more numerous than females at higher FXTAS stages (11.5% females vs. 88.5% males at stages 4 and 5). 

In contrast to the lack of significant differences recorded for WMHV in PFX− compared with non-carriers, PFX− and PFX+, both showed lower peripheral oxygen-linked ATP production sustained by succinate than non-carriers after age-adjustment (Table 1). Furthermore, within the premutation carriers, PFX+ exhibited higher oxygen-linked ATP production fueled by glycerophosphate than PFX− after age-adjustment (Table 1). These results may have two significant biological implications. First, it is possible that peripheral changes in mitochondrial bioenergetics associated with *FMR1* premutation are early changes of the disease that precede the development of WMHs in the brain and continue to accompany not only the onset but also the progression of FXTAS. This is consistent with the deficits in oxidative phosphorylation and reduced glucose metabolism shown as early pathological alterations in Alzheimer’s disease, another neurodegenerative condition [25,26]. Secondly, some of the subjects at higher FXTAS stages may be increasing the flux of fatty acids to offset the decline in ATP levels or production and/or overcome a deficit in the malate-aspartate shuttle. Although the higher values of oxygen-linked ATP production fueled by glycerophosphate by the PFX+ group compared with PFX− might seem beneficial, the fact that not all electron transport chain segments’ activities follow the same direction of change is more indicative of an altered protein handling (proteotoxicity), which is associated with impaired mitochondria-nuclear crosstalk [72] and mitochondrial unfolded protein response [73]. In addition, while in most tissues (including PBMCs) deficits in glucose metabolism via pyruvate dehydrogenase can be tolerated by utilizing other substrates to provide energy (e.g., glycerophosphate), this up-regulation might not improve brain energy homeostasis as fatty-acid oxidation in this organ is negligible [74,75].

The observation of a significant age-dependent decline in citrate synthase activity in PBMCs across carriers and non-carriers agrees with one of our earlier studies performed with a smaller number of participants (30 premutation carriers vs. 12 NCs; [23]). It is also consistent with another study showing a clear drop in citrate synthase activity in PBMCs between 16 and 20 and 46 and 55 years of age, remaining at a low, constant value through 66–89 years of age [76]. While in non-carriers the decrease in citrate synthase activity was mirrored by a decline in Complex IV activity (Spearman r = 0.6002, *p* = 0.0031) pointing to an overall decline in mitochondrial mass, no statistically significant correlation was observed between these outcomes in carriers (Spearman r = 0.1912 and 0.1040, *p* = 0.1542 and 0.2584 respectively for carriers without FXTAS and with FXTAS), suggesting a shrinkage of the mitochondrial matrix (or the TCA cycle) with respect to the electron transport chain.

Importantly, peripheral mitochondrial ATP production fueled by succinate or glucose-glutamine correlated negatively with brain WMHV (semi-partial r = −0.26 and −0.33, FDR = 0.03–0.08), and mitochondrial mass and succinate-fueled oxygen-linked ATP production correlated positively with BV (semi-partial r = 0.25–0.32, FDR = 0.03–0.08) when combining all premutation carriers (Table 2, Figure 2A,B). However, these correlations were no longer significant when age was included as a covariate implicating a critical role for age in mediating the associations between brain imaging findings and peripheral mitochondrial outcomes. This model also suggests that in patients with FXTAS, the decline in bioenergetics (function) is maintained across stages preceding the development of more detrimental phenotypes as indicated above.

The question of whether peripheral bioenergetic changes may reflect or be predictive of CNS mitochondrial deficits with links to WMHV and BV changes deserves a separate discussion. There is a clear overlap of features between premutation phenotype and mitochondrial diseases, including WMHs in both cerebral and cerebellar white matter in mitochondrial diseases and FXTAS [62,77,78]. Cerebellar atrophy, particularly affecting children, is another core feature of mitochondrial dysfunction [77]. Consistently, we have reported abnormal developmental trajectory of cerebellar volume in PFX− aged 8 to 81 years and significant cerebellar atrophy in PFX+ compared with both PFX− and NCs [65]. However, WMHs have not been observed in children with the premutation, suggesting that the detrimental effect of mitochondrial bioenergetic changes on the central nervous system (CNS) may not be as severe in children with the premutation relatively to children with mitochondrial disorders. However, both conditions (mitochondrial disorders and FXTAS) are clearly associated with energy deficits and cellular oxidative damage from reactive oxygen species [46]. Nonetheless, the current study provides direct evidence linking CNS imaging changes associated with the premutation phenotype represented by white matter damage and brain atrophy and reduced peripheral mitochondrial mass and ATP production. At the mechanistic level, our findings of mitochondrial dysfunction in the premutation may be explained by recent reports on fragile X syndrome’s models (contrary to the premutation, there is no detectable *FMR1* gene or FMRP protein expression in males and reduced FMRP expression in females), in which FMRP was found to regulate mitochondrial mRNA expression and energy homeostasis (murine model), and energy metabolism and mitochondrial function (*Drosophila* model) [79,80]. A recent study by our team provides further support for proteotoxicity and altered unfolded protein response at the core of the bioenergetic deficits in FXTAS [18,22,81,82], as sulforaphane-mediated normalization of these processes recovered mitochondrial function [82].

## 4. Materials and Methods

### 4.1. Research Participants

We included adults who participated in the genotype-phenotype study of families with fragile X from 2013 through 2019 with the availabilities of both MRI and blood samples (except for 7 non-carriers who did not undergo MRI). Written informed consent was obtained from all participants before participation in line with the Declaration of Helsinki. The study was approved by the Institutional Review Board of the University of California Davis Medical Center (Genotype-Phenotype Relationships in Fragile X Families, IRB Number 254134) and all methods were performed in accordance with their guidelines and regulations. FXTAS stage was scored by a trained physician (RJH) based on the severity of movement and gait impairments (stage 1: subtle or questionable signs, stages 2–6: clear tremor/balance problems with minor to severe interference of daily living) [61]. Premutation carriers at FXTAS stages ≤ 1 were combined into the non-FXTAS group (PFX−) whereas those at FXTAS stages ≥ 2 formed the FXTAS group (PFX+).

### 4.2. PBMCs Preparation

Blood (5–7 mL) was collected in BD Vacutainer Cell Preparation Tubes^TM^ (Becton-Dickinson, Franklin Lakes, NJ, USA) and processed according to the manufacturer’s recommendation within less than 1 h from blood collection. Most samples were collected between 9 and 11 a.m. Lymphocytes were isolated as previously described [22]. 

### 4.3. Mitochondrial Outcomes

Chemicals and biochemicals: EDTA, EGTA, sodium succinate, mannitol, sucrose, and HEPES were all purchased from Sigma (St. Louis, MO, USA). Tris-HCl, glycine, sodium chloride, and potassium chloride were purchased from Fisher (Pittsburg, PA, USA). Bovine serum albumin (fatty-acid free) was obtained from MP Biomedicals. All other reagents were of analytical or higher grade.

For polarographic determination of ATP-linked oxygen uptake of intact or permeabilized cells, we used a set-up of two Clark-type oxygen electrodes with two chambers [21,24,64,83,84,85,86,87,88]. The semipermeable membrane is changed the day before the experiment is planned to avoid unwanted cell debris that may have become attached to it. The membrane is hydrated a day before (for no less than 8 h) to facilitate oxygen diffusion. Washes of the chamber are done with 70% ethanol, and 3 washes of dd water. The calibration of the electrode entails the recording of zero oxygen concentration (with dithionite) and air-saturate solution (used for functional studies) warmed up at the temperature at which the experiments are run. The calibration is run in duplicates with <10% CV. The oxygen concentration in the calibrating solution is calculated with the atmospheric pressure (barometer) and ambient temperature (thermometer). Additions to the chamber are done by using Hamilton syringes to avoid increasing oxygen concentrations throughout the evaluations. The chamber is constantly stirred with a Teflon-coated minibar to ensure a homogenous diffusion of substrates and oxygen. Washes with 70% ethanol are warranted after using rotenone, antimycin or FCCP, which tend to stick to the plastic walls of the chamber. ATP-driven oxygen uptake is usually done in duplicates at a given cell concentration (which was calculated before starting this protocol). All enzymatic assays are performed within the hour of collecting the blood sample and run in parallel with controls. Reproducibility is ensured by running a subset of samples previously tested in parallel with new batches of samples.

Activities of Complexes I–V in digitonin-permeabilized lymphocytes were determined by polarography essentially as described before [22,88]. Briefly, an aliquot (1.0–2.0 × 10^6^) of lymphocytes was added to the chamber equipped with a Clark-type Hansatech oxygen electrode at 20–22 °C in 0.3 mL of buffer containing 0.22 M sucrose, 50 mM KCl, 1 mM EDTA, 10 mM KH_2_PO_4_, and 10 mM HEPES, pH 7.4. Oxygen consumption rates were evaluated in air-saturated solutions in the presence of (i) 1 mM ADP plus 1 mM malate-10 mM glutamate followed by the addition of 5 μM rotenone; (ii) 10 mM succinate followed by the addition of 1 mM malonate; (iii) 1 mM α-glycerophosphate followed by the addition of 3.6 μM antimycin A; and (iv) 10 mM ascorbate and 0.2 mM *N,N,N*′*,N*′-tetramethyl-*p*-phenylenediamine followed by the addition of 1 mM KCN (activity of Complex IV). Activities of individual electron transport chain (ETC) segments were evaluated as the difference of oxygen uptake recorded before and after the addition of specific inhibitors. Most mitochondrial inhibitors and uncouplers were stored at −80 °C as concentrated stock solutions (high mM) in DMSO to prevent unwanted oxidation or degradation. Quality control checks were performed with beef heart submitochondrial particles and results were compared to data collected over the years.

Oxygen consumption was also evaluated in intact cells using a Clark-type oxygen electrode (Hansatech, King’s Lynn, UK) as previously described [23,89]. ATP-linked oxygen uptake (or State-3-dependent oxygen uptake) was calculated as the difference between basal and oligomycin-induced State 4 oxygen uptake rates; State 4o is the residual respiration after inhibition of ATP synthesis with the ATPase-specific inhibitor 0.2 µM oligomycin; maximal respiratory capacity, or State 3u, is described as the oxygen uptake rate in the presence of 2 µM of the uncoupler carbonyl cyanide-4-(trifluoromethoxy) phenylhydrazone (FCCP); respiratory control ratio (RCR) was calculated as the ratio between States 3 and 4o; index of respiratory capacity (IRC) was calculated as the difference between State 3 and State 4o normalized by that of State 3u. Mitochondrial proton leak (PL)/ROS production was calculated from the oligomycin-resistant oxygen consumption rates and normalized by basal respiration in the presence of 10 mM glucose-2 mM glutamine in RPMI-1640.

Citrate synthase activity was evaluated spectrophotometrically with a Tecan Infinite M200 microplate reader at 412 nm as described before by using 2.5 to 3 × 10^5^ cells [22]. All cell pellets destined for this activity were tested within the hour of blood extraction. If stored, the pellets were supplemented with proteolytic inhibitors (4-benzenesulfonyl fluoride hydrochloride, EDTA, bestatin, E-64, leupeptin, aprotinin, from Sigma) and kinase and phosphatase inhibitors (sodium orthovanadate, sodium molybdate, sodium tartrate, imidazole, cantharidin, (-)*p*-bromolevamisole oxalate, calyculin A, from Sigma) and stored at −80 °C.

### 4.4. MRI Acquisition and Processing

MRI scans were acquired on a Siemens Trio 3T MRI scanner equipped with a 32-channel head coil (Siemens Medical Solutions, Erlangen, Germany). One-millimeter isotropic T1-weighted scans were collected covering the whole brain using the magnetization prepared rapid gradient-echo (MPRAGE) sequence in 192 sagittal slices with repetition time (TR) of 2170 ms, echo time (TE) of 4.82 ms, and 7° flip angle. Fluid attenuated inversion recovery (FLAIR) images for quantifying WMHV were acquired in 104 sagittal slices of 1.9-mm thickness with an in-plane resolution of 0.47 mm^2^, TR of 5000 ms, TE of 456 ms, and inversion time 1700 ms.

Both T1 and FLAIR scans were corrected for intensity inhomogeneities due to MRI bias field using N4 [90]. BV and brain scaling factor (for correcting individual differences in cranial size) were obtained on MPRAGE scans using the SIENAX program [91] from FSL. Optimal values were obtained by adjusting the parameter used for brain extraction. WMHV was quantified on FLAIR images using lesion prediction algorithm from SPM12 [92]. Lesion masks were generated by setting appropriate thresholds on lesion probability maps using the FSL command, fslmaths, followed by manual correction for errors using ITK-Snap [93]. WMHV calculations were performed using the FSL command, fslstats.

### 4.5. Statistical Analysis

All statistical analyses were conducted in the open-source statistical package R 3.6.3 (http://www.r-project.org/ accessed on 25 June 2020). Multiple linear regression was used to study the effects of age, group, sex, and age-by-group interaction on individual MRI/mitochondrial outcomes. Contrasts were used to make specific group comparisons within a regression model. WMHV and most mitochondrial outcomes (except the index of respiratory capacity and respiratory control ratio; IRC and RCR) did not follow normal distributions in premutation carriers. Consequently, log-transformation was applied to these variables before performing any statistical analyses. Since FXTAS commonly occurs in premutation carriers aged 50 and above, the correlations between FXTAS stage and MRI/mitochondrial outcomes were analyzed including only the premutation carriers older than 50 years, while controlling for age and sex. Brain scaling factor was added as a covariate for all statistical models involving the MRI data. Model selection procedure was carried out using a stepwise approach via the likelihood ratio test. The correlations between MRI and mitochondrial measurements were examined in all premutation carriers. Semi-partial correlation coefficients from the R package “ppcor” were employed to assess the associations between individual mitochondrial outcomes and individual MRI measures while controlling cranial size for MRI measures only. The Benjamini-Hochberg method of false discovery rate (FDR) [94] was applied to control the FDR at 10% for all hypotheses tested in one type of analysis.

## Figures and Tables

**Table 1:** Effect of age and FXTAS stage diagnoses on MRI outcomes and peripheral mitochondrial bioenergetics.

Measures	*N*	Age			PFX− vs. Controls			PFX+ vs. Controls			PFX+ vs. PFX−		
		*β* (SD)	*P*	FDR	β ** (SD)	*P*	FDR	β ** (SD)	*P*	FDR	β ** (SD)	*P*	FDR
*MRI*													
WMHV (log)	96	0.050 (0.011)	<0.001	**<0.001**	0.005 (0.45)	0.99	0.99	1.54 (0.52)	0.004	**0.007**	1.53 (0.36)	<0.001	**<0.001**
BV (ml)	96	−3.18 (0.62)	<0.001	**<0.001**	−52.2 (24.4)	0.035	**0.046**	−86.8 (28.6)	0.003	**0.007**	−34.7 (19.6)	0.08	**0.09**
*Mitochondria*													
CS *	103	−0.026 (0.006)	<0.001	**<0.001**	−0.46 (0.24)	0.06	0.18	−0.30 (0.23)	0.20	0.39	0.16 (0.22)	0.45	0.61
NADH-dep. ATP prod. *	102	−0.021 (0.006)	0.001	**0.008**	−0.47 (0.23)	0.045	0.16	−0.25 (0.22)	0.26	0.46	0.22 (0.21)	0.30	0.47
FADH_2_-dep. ATP prod. *	103	−0.027 (0.006)	<0.001	**0.001**	−0.57 (0.24)	0.020	**0.10**	−0.55 (0.23)	0.020	**0.10**	0.03 (0.22)	0.91	0.94
GP *	97	−0.032 (0.007)	<0.001	**<0.001**	−0.62 (0.28)	0.029	0.12	0.012 (0.27)	0.97	0.97	0.64 (0.26)	0.016	**0.10**
CCO *	102	−0.035 (0.008)	<0.001	**<0.001**	−0.22 (0.21)	0.31	0.47	−0.44 (0.22)	0.051	0.17	−0.22 (0.21)	0.20	0.39
Basal *	95	−0.031 (0.008)	<0.001	**<0.001**	−0.52 (0.30)	0.08	0.23	−0.17 (0.29)	0.55	0.68	0.35 (0.27)	0.19	0.39
RCRu *	95	−0.003 (0.005)	0.51	0.65	−0.27 (0.18)	0.14	0.35	−0.38 (0.18)	0.03	0.13	−0.11 (0.16)	0.49	0.64
SRC *	95	0 (0.004)	0.92	0.94	0.03 (0.16)	0.85	0.93	−0.14 (0.15)	0.35	0.51	−0.17 (0.14)	0.22	0.41
PL/ROS *	95	0.001 (0.005)	0.88	0.94	0.27 (0.20)	0.19	0.39	0.31 (0.20)	0.12	0.33	0.04 (0.18)	0.83	0.93
IRC	95	0.001 (0.003)	0.69	0.79	−0.09 (0.10)	0.35	0.51	−0.13 (0.10)	0.16	0.38	−0.04 (0.09)	0.64	0.75
RCR	95	−0.006 (0.007)	0.38	0.53	−0.30 (0.29)	0.30	0.47	−0.43 (0.28)	0.13	0.33	−0.13 (0.26)	0.62	0.75

In bold, significant with FDR ≤ 0.10. BV, brain volume; WMHV, white matter hyperintensity volume; CS, citrate synthase; NADH-dep. ATP prod., NADH-dependent ATP production; FADH_2_-dep. ATP prod., FADH_2_-dependent ATP production; GP, α-glycerophosphate-sustained ATP production; CCO, cytochrome *c* oxidase; basal, glucose-Gln-sustained ATP production; RCRu, respiratory control ratio under uncoupling conditions; SRC, spare respiratory capacity; PL/ROS, proton leak/reactive oxygen species; IRC, index of respiratory capacity; RCR, respiratory control ratio. *, indicates the application of log-transformation prior to statistical analysis. **, presents contrast coefficients of the group comparisons in the regression models.

**Table 2:** Semi-partial correlations between mitochondrial outcomes and volumes of WMHs and whole brain in premutation carriers controlling cranial size in MRI data.

Mitochondrial Outcomes	*N*	Semi-Partial *r*	*P*	FDR	*N*	Semi-Partial *r*	*P*	FDR
With log white matter hyperintensity volume					With whole brain volume			
Citrate synthase activity *	87	−0.227	0.036	0.13	87	0.249	0.021	**0.08**
NADH-linked ATP production *	86	−0.172	0.115	0.25	86	0.158	0.149	0.33
FADH_2_-linked ATP production *	87	−0.264	0.014	**0.08**	87	0.246	0.023	**0.08**
Glycerophosphate-linked ATP production *	79	−0.091	0.427	0.67	79	0.064	0.576	0.63
Cytochrome oxidase activity *	86	−0.176	0.107	0.25	86	0.323	0.003	**0.03**
Glucose-Gln-fueled ATP production *	81	−0.330	0.003	**0.03**	81	0.213	0.058	0.16
RCRu *	81	−0.023	0.842	0.84	81	0.098	0.385	0.61
SRC *	81	−0.105	0.354	0.65	81	0.020	0.862	0.86
PL/ROS *	81	−0.040	0.727	0.84	81	−0.070	0.540	0.63
IRC	81	0.026	0.819	0.84	81	0.082	0.470	0.63
RCR	81	−0.060	0.599	0.82	81	0.135	0.231	0.42

In bold, FDR ≤ 0.10. *, indicates the application of log-transformation prior to statistical analysis. Abbreviations: see under Table 1.

## Data Availability

Data presented in this study are contained within the article or Supplementary Material.

## Supplementary Materials

The following are available online at https://www.mdpi.com/article/10.3390/ijms22179171/s1.
