# Supplementary material for: Brain Atrophy and White Matter Damage Linked to Peripheral Bioenergetic Deficits in the Neurodegenerative Disease FXTAS
Source: Int J Mol Sci. 2021 Aug 25;22(17):9171. doi: 10.3390/ijms22179171 (PMC8431233; doi:10.3390/ijms22179171)
Supplement: Supplementary file 1 [file ijms-22-09171-s001.zip › ijms-1305700-supplementary.pdf]

Supplementary Material

Brain atrophy and white matter damage linked to peripheral bioenergetic deficits in the neurodegenerative disease FXTAS, by Wang/Napoli, et al.

Table S1. Correlations between WMHV, BV, peripheral mitochondrial outcomes and FXTAS stage adjusted for sex**

Outcome	*β*	SD	*P*-Value
WMHV	0.439	0.101	< 0.001
BV	-2.706	2.050	0.191
Log citrate synthase activity	0.025	0.178	0.888
Log NADH-linked ATP production*	-0.081	0.189	0.671
Log $FADH_2$-linked ATP production	-0.150	0.156	0.340
Log glycerophosphate-linked ATP production	-0.052	0.151	0.734
Log cytochrome *c* oxidase activity	-0.252	0.201	0.214
Log glucose-Gln-fueled ATP production	-0.006	0.134	0.965
Log RCRu	-0.463	0.260	0.079
Log SRC	-0.403	0.293	0.173
Log ROS/PL	0.219	0.239	0.362
IRC	-0.296	0.487	0.545
RCR	-0.266	0.173	0.129

*ATP production stands for oxygen-linked ATP production.

** All abbreviations were spelled out in the main text and Methods.
